# Dissociable effects of practice variability on learning motor and timing skills

**DOI:** 10.1371/journal.pone.0193580

**Published:** 2018-03-01

**Authors:** Baptiste Caramiaux, Frédéric Bevilacqua, Marcelo M. Wanderley, Caroline Palmer

**Affiliations:** 1 UMR STMS Ircam-CNRS-UPMC, Paris, France; 2 Schulich School of Music, McGill University, Montreal, Canada; 3 Department of Psychology, McGill University, Montreal, Canada; 4 CNRS, LRI, Univ. Paris-Sud, University Paris-Saclay, Inria, Gif-sur-Yvette, France; University of Western Ontario, CANADA

## Abstract

Motor skill acquisition inherently depends on the way one practices the motor task. The amount of motor task variability during practice has been shown to foster transfer of the learned skill to other similar motor tasks. In addition, variability in a learning schedule, in which a task and its variations are interweaved during practice, has been shown to help the transfer of learning in motor skill acquisition. However, there is little evidence on how motor task variations and variability schedules during practice act on the acquisition of complex motor skills such as music performance, in which a performer learns both the right movements (motor skill) and the right time to perform them (timing skill). This study investigated the impact of rate (tempo) variability and the schedule of tempo change during practice on timing and motor skill acquisition. Complete novices, with no musical training, practiced a simple musical sequence on a piano keyboard at different rates. Each novice was assigned to one of four learning conditions designed to manipulate the amount of tempo variability across trials (large or small tempo set) and the schedule of tempo change (randomized or non-randomized order) during practice. At test, the novices performed the same musical sequence at a familiar tempo and at novel tempi (testing tempo transfer), as well as two novel (but related) sequences at a familiar tempo (testing spatial transfer). We found that practice conditions had little effect on learning and transfer performance of timing skill. Interestingly, practice conditions influenced motor skill learning (reduction of movement variability): lower temporal variability during practice facilitated transfer to new tempi and new sequences; non-randomized learning schedule improved transfer to new tempi and new sequences. Tempo (rate) and the sequence difficulty (spatial manipulation) affected performance variability in both timing and movement. These findings suggest that there is a dissociable effect of practice variability on learning complex skills that involve both motor and timing constraints.

## Introduction

Motor learning research has shown for simple tasks that varying a motor task during practice can facilitate motor adaptation and the learning of a new skill [1–5]. An important benefit of movement variability during motor skill acquisition is the facilitation of transfer to novel motor tasks. In other words, by practicing several variations of a motor task, one can more effectively perform other motor tasks that share structural similarities [1]. This benefit is consistent with the notion of motor schemas [2]. A schema is an abstraction of the relationships between motor commands and sensory consequences of the command underlying a given motor skill. The strength of a motor schema is its potential for generalization, which means the transfer to novel motor tasks. In a seminal work, Schmidt [2] argued that variability in practice improves the strength of a schema. This theory has been tested in implicit learning contexts [6], applied to sports, like underhand volleyball serve [7], and musical instrument technique, such as wide left-hand interval leaps [8].

It is not yet clear how the benefit of motor task variations in skill acquisition and transfer of learning applies to complex movement sequences and, especially, timed movement sequences. Timed movements are particularly important in music performance in which sequences of movements must be performed both in the right order and at the right time [9]. In piano performance, for instance, a movement may involve complex coordination of finger movements under biomechanical and cognitive constraints between fingers [10,11]. Such constraints must be integrated during practice with the ability to perform at the right tempo. Thus, the contribution of task variations to the improvement of timing and motor skills during practice of timed movements is largely unknown.

Motor variability during musical practice has been examined in a few studies with mixed findings. Motor variability was examined as college music students (with a minor in piano) learned a large left-hand interval on a piano keyboard [8] under three conditions: a fixed practice where only one interval was learned (the target interval); a variable practice where 4 intervals were learned, including a target interval; and “spaced” practice where only one interval was learned (the target interval) for the same number of trials as in the variable practice group, spaced among the same duration as for the fixed and variable groups. Performance was tested on the target interval after training and re-tested 24h after practice (retention). Transfer performance was tested on a novel interval after practice and at retention. The findings showed that all groups performed equivalently (with similar error rates) on the original target interval. In addition, fixed practice led to significantly more errors (wrong notes played) at transfer to the novel interval than did spaced practice and variable practice. At retention though, only the variable practice showed a significant difference between the target interval and transfer to the novel interval, with more errors for the transfer interval. These results thus suggest that over-practicing leads to poorer transfer right after training, but variability of practice leads to poorer transfer at retention [8].

Another factor that influences motor learning during practice is *contextual interference* [4,12]: a learning schedule in which a task and its variations are interweaved, and the control variable is the time spent on each task variation. The idea is that injecting contextual interference by frequently alternating motor tasks results in increased long-term learning that is more likely to transfer to new tasks [12]. Contextual interference has applications in sports [13] and handwriting [14] but has received only little attention in music performance [15–18] where blocked repetition of a musical task, that is, the practice of one task up to satisfactory acquisition before switching to another, remains the most common practice schedule [15,17]. Stambaugh [17] compared the effect of blocked and random practice conditions in learning three clarinet examples over a 3-day period. In the blocked condition, student clarinetists performed one example for 18 trials on each day, while in the random condition, students performed six trials of each of the 3 examples in a random order on each day. Acquisition was tested on the last three trials of the last day, retention was tested 24h after the last day and transfer test was tested after retention. The effects of both practice conditions were assessed on several dependent variables: clarinetists' performance accuracy, speed, temporal evenness, and attitude. The author found a difference only in speed between practice schedules: the random condition group played faster without losing in accuracy at retention (but not at transfer). No other differences were found in dependent variables between practice conditions.

In a recent study, Carter and Grahn [15] studied the impact of blocked and interleaved practice schedules on expert clarinet performance in a real-world context. Expert clarinetists started by learning a first concerto exposition and a technical excerpt 12min each (blocked condition), and then learned a second concerto exposition and a second technical excerpt 3min each, alternating until having practiced each piece 12min (interleaved condition). Performances at the end of both practice sessions as well as performances played one day after practices were recorded and analyzed by professional clarinetists (raters). The findings showed that some raters perceived differences in concerto expositions and technical exercises when these pieces were practiced in the interleaved schedule. However, there was no significant perceived differences between pieces when the scores were averaged across raters, suggesting that contextual interference did not systematically improve learning.

It is not clear from these studies if the relatively small impact of the amount and the schedule of task variability during musical practice is related to the music-specific task complexity, which involves both motor and timing skills that may have different effects on the dependent variables considered in these studies. Switching from one musical excerpt to another requires both spatial variations (different pitches to play) and temporal variations (different rhythms and rates). In addition, spatial variations may involve various levels of motor complexity related to finger coordination. Here we focus on one type of task variation, musical rate or tempo variation, and we hypothesize that this type of task variation does not act equivalently on the acquisition of motor skills and timing skills. Timing skill is defined here as the capacity to strike a piano key at the right time (considering a given tempo) and motor skill is defined as the capacity for reduced movement variability (smoother movement) as fingers move between two successive piano keys.

We hypothesize that tempo variations during musical practice will influence motor learning: significant differences in movement smoothness under different tempo practice conditions will yield different transfer for complex musical stimuli as reported in previous work on motor learning [4]. In contrast, we hypothesize that tempo variations during practice will not necessarily influence timing regularity at test, as timing in discrete motor tasks, including music performance, has been shown to be explicit and independent from movement production [19,20]. Importantly, effects of tempo variability at practice and contextual interference have generated different effects at immediate test and at retention [4,12]; we inspect differences in the effect of tempo variations during practice on timing and motor skill learning at immediate tests after training.

To test this hypothesis, we investigated the impact of the amount of tempo variability and the schedule of tempo change on timing skill acquisition and motor skill acquisition during the practice of a timed sequence performed at the piano. An important and novel goal was to study complete musical novices in the piano performance task in order to remove the potential influence of prior skills or practice habits due to training. The novices practiced a short musical sequence on many trials on a piano keyboard at different rates indicated by an audio sample. Each novice was assigned to one of four learning conditions designed to manipulate the amount of tempo variability across trials (large or small tempo set) and the schedule of tempo change (randomized order or non-random order) during practice. At test, the novices performed the same musical sequence at a familiar tempo and at novel tempi (testing tempo transfer), and two novel (but related) sequences at a familiar tempo (testing motor complexity transfer through spatial manipulations). Timing skill acquisition was assessed through the reduction of timing variability and the increase of timing accuracy, measured from the pianists' keystrokes. Motor skill acquisition was assessed through the reduction of movement variability, measured by movements of the participants' finger joints during the movement execution. The experimental paradigm differs from that of motor sequence learning tasks (see for instance [21]) in that learning is assessed in terms of timing and motor quantitative measures, rather than in error rates in sequence production.

## Method

### Participants

Forty-eight participants were recruited (25 Female, M = 22.5 years old, SD = 3.4, age range = 18?34) from the McGill community. All participants were right-handed and none reported any neurological conditions. Recruited participants were all non-musicians, meaning they had never undertaken any courses or private lessons to learn how to play a musical instrument, and they had never learned an instrument by themselves. 12 participants were assigned randomly to each of the 4 test groups described below: Small Tempo Set–Non-Randomized; Small Tempo Set–Randomized; Large Tempo Set–Non-Randomized; Large Tempo Set–Randomized. The mean age of the 4 test groups did not differ (M_1 = 22.3, SD = 2.8; M_2 = 21.9, SD = 2.9; M_3 = 22.9, SD = 3.3; M_4 = 22.9, SD = 4.4). Participants gave informed consent prior to starting the experiment. The experiment was reviewed by the Research Ethics Board Office of McGill University (12–0616).

### Stimulus materials and equipment

**Fig 1:**
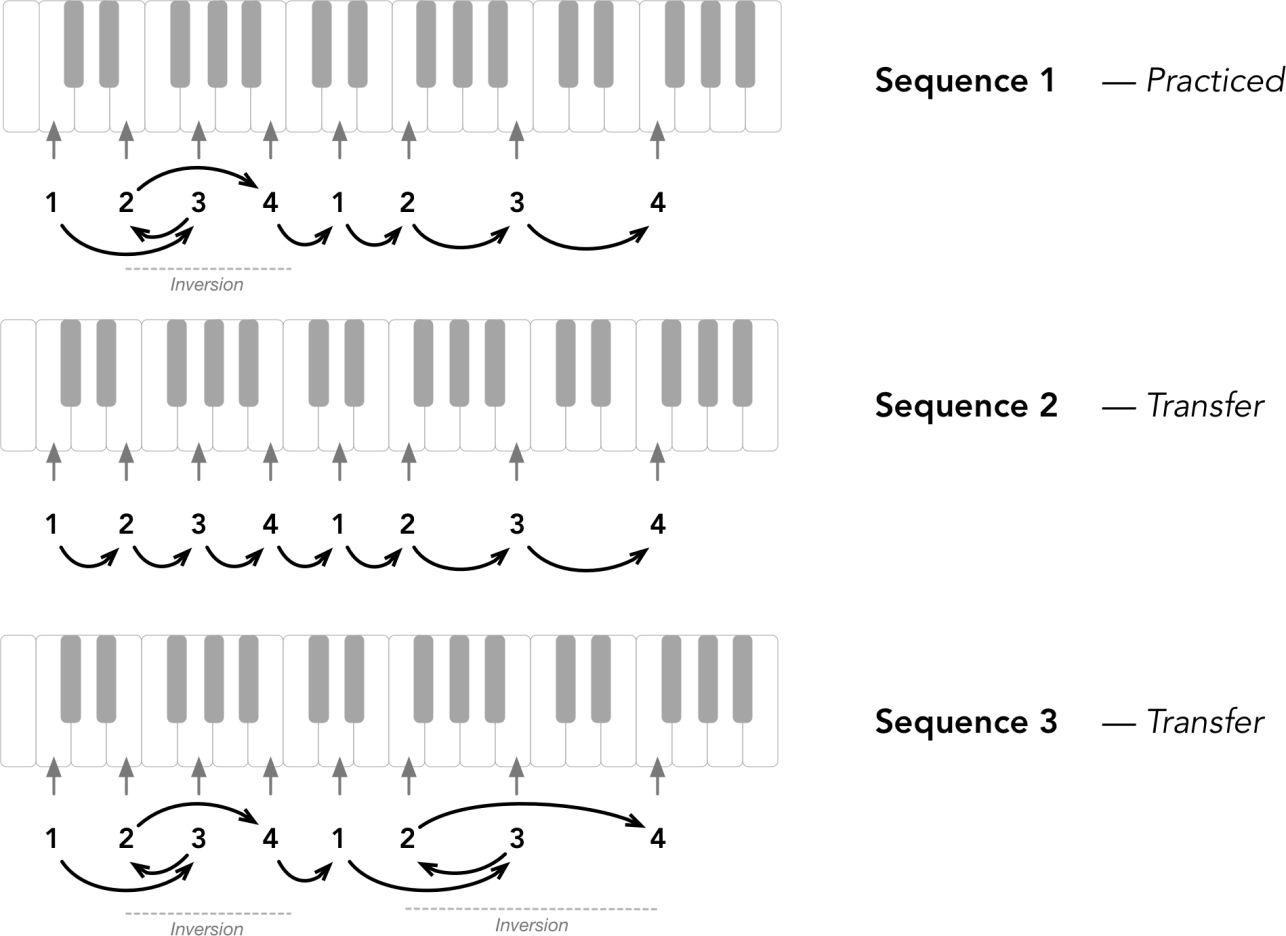
Music sequences of eight tones to be performed with one hand on the piano keyboard. Top panel is the main sequence (sequence 1) with 8 pitches and the finger assignments. Each finger was associated with a color marked with colored tape on the keyboard. Two alternative transfer sequences were also created: Sequence 2 (middle panel) involves the sequence of fingers F1-F2-F3-F4-F1-F2-F3-F4, that is inverting the second and the third notes from the original sequence; and Sequence 3 (bottom panel) involves the sequence of fingers F1-F3-F2-F4-F1-F3-F2-F4, that is inverting notes 6 and 7 from the original sequence.

A short musical sequence of eight tones to be performed with one hand on the piano keyboard was created for the study. The pitch sequence and the finger assignments are shown in the top panel of Fig 1. A specific sequence of fingers was associated to the pitches, given by: 1-3-2-4-1-2-3-4, where 1 denotes the thumb of the right hand, 2 is the index finger, 3 is the middle finger and 4 is the ring finger. The sequence was designed to use each finger equally often (2 times each), and to involve an *inversion* which is when two elements of the sequence swap orders, specifically fingers 2 and 3 within the sequence of fingers 1-2-3-4. Therefore Sequence 1 has an inversion in the first half of the sequence. Each pitch in the sequence was marked on the piano keyboard with colored tape that indicated to participants which finger to use, with a single color associated with each finger: *green* for thumb, *pink* for index, *yellow* for the middle finger and *blue* for ring finger.

Auditory recordings of the melody were generated at different rates defined by Inter-Onset Intervals {200ms, 250ms, 300ms, 350ms, 400ms, 450ms, 500ms, 550ms} (which in musical notation correspond to 300bpm (beat per minute), 240bpm, 200bpm, 171bpm, 150bpm, 133bpm, 120bpm, 109bpm respectively) to represent the range of tempi presented in the experiment as detailed below. Auditory recordings were generated with the grand piano timbre of the Yamaha CP300 digital keyboard used in the study. Each pitch within the melody was generated at the same loudness level given by a constant MIDI velocity control set to 60.

Transfer learning was tested with two alternative sequences, each one involving a unique modification from the original sequence as depicted in Fig 1 (middle and bottom panels). The first transfer sequence, denoted Sequence 2 (middle panel on the Figure) removes the inversion in the original sequence, resulting in the following sequence of fingers for the first 4 notes: 1-2-3-4 instead of 1-3-2-4. The second transfer sequence, denoted Sequence 3 (bottom panel on the Figure), inverts notes 6 and 7 from the original sequence, resulting in the following sequence of fingers: 1-3-2-4-1-3-2-4. Sequences 2 and 3 were equally different from Sequence 1. However, Sequence 3 Fig 1, bottom panel) is expected to be more difficult to perform because it involves two inversions: the original inversion in the first half of the sequence and a second inversion in the second half.

Participants performed the melody on the Yamaha CP300 digital keyboard. The MIDI and mono audio outputs from the keyboard were sent through two separate cables to an audio interface (RME Fireface 400) connected to a MacBook Pro computer (13-inch, 3.1GHz, MacOS 10.11) by Firewire. A custom-made program, implemented in the Max/MSP programming environment by Cycling 74, was used to record both signals as follows. The mono audio signal was captured at 48Hz and stored as the first channel of the audio file. MIDI velocities and pitches of keyboard information were independently converted to audio signals at 48Hz and stored as two additional audio channels of the same audio file. This process leads to two piecewise constant signals. We ensured synchronization between audio and MIDI through a global synchronization unit that generated time codes every 40μs (Rosendahl Nanosyncs HD). The generated synchronous signal (SMPTE timecode) was also recorded as an additional audio channel with the audio and midi data, within the custom-made software. A speaker (Genelec 8030A) was placed in front of the piano keyboard and the auditory recordings as well as the piano keyboard's output were sounded over this speaker.

**Fig 2:**
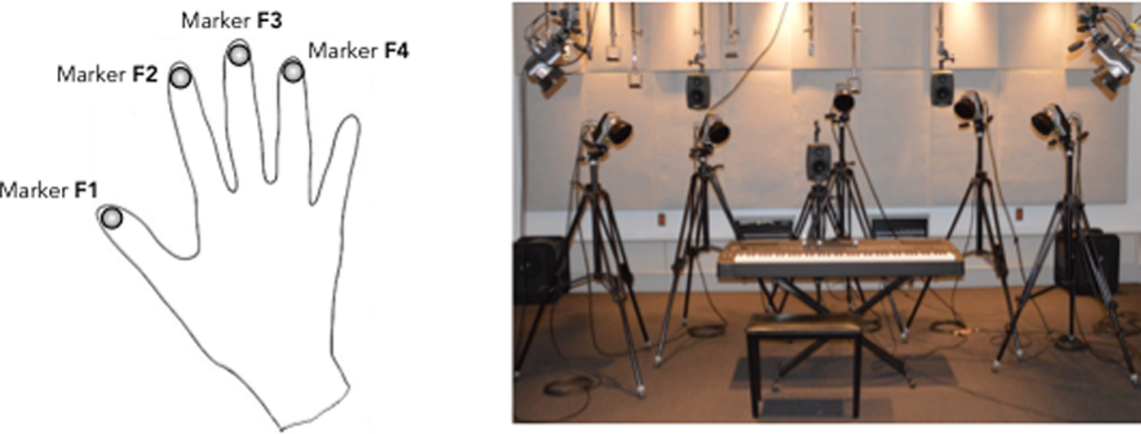
Motion capture markers and set-up. The left panel depicts the 4 markers placed on the participant's right hand. One marker is placed on each finger. The right panel depicts the configuration of the cameras and the speaker around the piano keyboard.

Participants' hand movements were captured with a passive motion capture system (Qualysis) which recorded reflected light from a set of 4 markers placed at the finger end-point. Fig 2 (left panel) depicts the placement of the 4 markers on the right hand of a participant. We used 8 infra-red cameras (Qualysis Opus 400) placed around the piano keyboard to record the markers' displacement during performance (see Fig 2, right panel). The motion capture data were recorded on a second computer (Windows 7) with Qualysis' proprietary software, at a sampling rate of 240Hz. To ensure synchronization between motion data and both audio and MIDI, we used the same SMPTE timecode as used for the audio and the MIDI signals to control the sample rate of the motion capture software.

### Design

The experiment was divided into 2 primary phases: a practice phase and a test phase. The practice phase employed a 2x2 between-subjects design with repeated measures. Two independent variables of practice were manipulated between subjects in the practice phase: The size of the tempo set (Small IOI {350ms, 450ms}, or Large IOI {250ms, 300ms, 350ms, 450ms, 500ms, 550ms}) at which participants practiced the melody; and the sequence Order (Random or Non-random) of the tempi to which they were exposed. All participants performed 6 successive trials at the same tempo per practice block.

**Fig 3:**
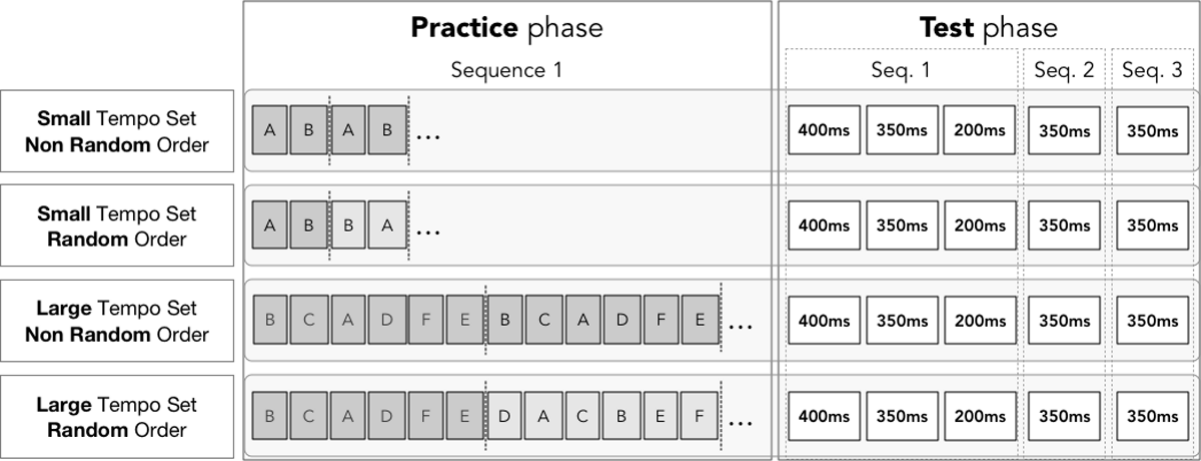
The study procedure, depicting the practice phase and a test phase. A, B, C, D, E, F indicate practice tempi. For groups 1 and 2, A = 350ms and B = 450ms. For groups 3 and 4, A = 250ms, B = 300ms, C = 350ms, D = 450ms, E = 500ms, F = 550ms.

In the Non-Randomized condition, a permutation of the tempos from the Small or Large Tempo Set was created for the first set of practice blocks and was then repeated throughout the experiment. An example is shown in Fig 3. In the Randomized condition, the tempo order was freshly permuted for each set of practice blocks, as shown in Fig 3. There was a total of 144 practice trials: in the Small Tempo Set condition, both tempi are practiced 72 times (12 practice blocks of 6 trials for each tempo); in the Large Tempo Set, the 6 tempi are practiced 24 times (4 practice blocks of 6 trials for each tempo).

At the test stage, participants performed the melody for 12 trials in each of 3 tempi: 400ms IOI (a novel tempo), 350ms IOI (a practiced tempo), and 200ms IOI (another novel tempo, fast). The order of tempo conditions was kept constant. Finally, participants performed the transfer sequences (see Fig 1), at tempo 350ms IOI, for 12 trials each. There was a total of 60 test trials.

### Procedure

First, each participant was told that (s)he will learn to play a melody at the piano by repeating the melody they heard over a speaker at different tempi. Prior to starting, the experimenter placed the set of 19 motion capture markers on the participant's right hand. The participant was told that these markers are used to capture the movements of the hand through the cameras placed around the keyboard. Each participant was then invited to sit in front of the keyboard and participated in a warm-up task. During the warm-up phase, participants were told that they would hear an audio recording of the melody; once the melody ended they performed the melody on the keyboard, at the same tempo, being as regular as possible in tempo. They were shown the keys marked on the keyboard to play the melody, and they were told the sequence of fingers to use to perform the sequence. Then they were given three trials at a tempo of 450ms per tone to practice the task. A trial in each phase of the experiment was defined as: 1) listening to the auditory recording of the sequence at a fixed tempo, and 2) performing the sequence on the piano at the tempo of the auditory recording once the recording ended.

During the practice phase, participants were told that the task will be the same, but the tempo of the audio recording of the melody will change. They were given 6 practice trials at each tempo and they performed 144 trials total.

During the test phase, participants were told they would perform 12 trials at each tempo and that they would perform at 3 different tempi. There was a 1-minute break between each tested tempo. Then they were told that they would perform 12 trials at a single tempo but with a modification to the finger movement sequence (Transfer Sequence 2, Fig 1, middle panel). The new sequence was explained to them and the 12 trials started without rehearsal. Finally, they were told that they would perform 12 trials at a single tempo but with an additional modification in the finger movement sequence (Transfer Sequence 3, Fig 1, bottom panel). Thus, sequence 2 was designed to be easier than sequence 1, and sequence 3 was designed to be more difficult than sequence 1. Similarly, no rehearsal was allowed before testing. There were 60 trials in all in the test phase.

The entire experiment lasted 1 hour and participants received a nominal fee for their participation.

### Data analysis

#### Timing analysis

Both practice and test trials were assessed by the accuracy and variability of keypress Inter-Onset Interval (IOI) timing [22]. The timing variability within each performance trial was assessed with the coefficient of variation (CV) of the IOIs (in milliseconds) within a single trial [11], defined as the standard deviation divided by the mean of the IOIs.

The timing accuracy was defined by the deviation from the expected tempo (tempo of the stimulus audio recording heard prior to each performance), assessed by the observed IOIs minus the expected IOIs for each tone within a single trial, divided by the expected IOIs. Positive values indicate slowing (longer intervals) in the sequence while negative values indicate rushing (shorter intervals). The analyses consider the mean signed timing deviations for each trial.

#### Movement analysis

Complementary to timing analysis, we analyzed the movement kinematics between the tone onsets in order to assess the acquisition of motor skills. The finger-motion data were first translated relative to the plane of the piano keyboard such that x corresponds to the side-to-side dimension along the piano keys (positive values are rightmost pitches), y corresponds to forward-backward dimension (positive y values are forward movements), and z corresponds to the vertical dimension (positive values are upward movements). To assess the amount of skill in terms of execution quality, typically understood as smoothness [23], we measured the jerk [24], the third derivative of position, for each finger relative to the keypresses during sequence production. The motion data from each trial was segmented on the basis of the MIDI key onsets. Within each IOI, the motion data of the fingertip that pressed the next key was examined. For each fingertip keystroke's motion segment, we computed the squared jerk, which accounts for the rate of change in the movement acceleration, and we integrated this quantity along the segment trajectory [25]. Squared jerk integration was performed along the three dimensions *(x*,*y*,*z)* in order to take into account the smoothness of the global movement and not only along a given axis. Jerk from the *(x*,*y*,*z)* position time series of the fingertips was computed using a Savitzky-Golay derivative filter [26] with a cut-off frequency of 19.3Hz. This process yielded one smoothness measure per Inter-Onset per trial.

We computed a smoothness criterion as the mean of the integrated squared jerks across Inter-Onsets divided by its standard deviation. The rationale behind dividing the mean by the standard deviation is to be able to compare the smoothness criterion across participants since we are expecting high inter-participant variability.

## Results

The number of incorrectly performed sequences (defined as an incorrect sequence of piano keystrokes) was 391 out of 6912 practice trials (5.7%); 167 out of 1728 test trials of sequence 1 (9.7%); 40 out of 576 test trials of sequences 2 (6.9%) and 46 out of 576 of sequences 3 (8.0%). The number of incorrectly performed sequences did not differ across practice conditions (number of different tempi and ordering of tempi during practice) and were excluded from subsequent analyses. We also assessed whether there was any initial performance bias between groups due to participants' individual prior skills. Mean temporal variability measures from the first practice trial in each condition were compared with a two-way analysis of variance with Tempo Set (2 or 6) and Tempo Order (Random or non-Random). We found no significant differences for Tempo Set (F(1,44) = 0.29, p = 0.59) or for Order (F(1,44) = 0.021, p = 0.89), indicating that the groups did not differ at the beginning of the experiment.

### Practice

**Fig 4:**
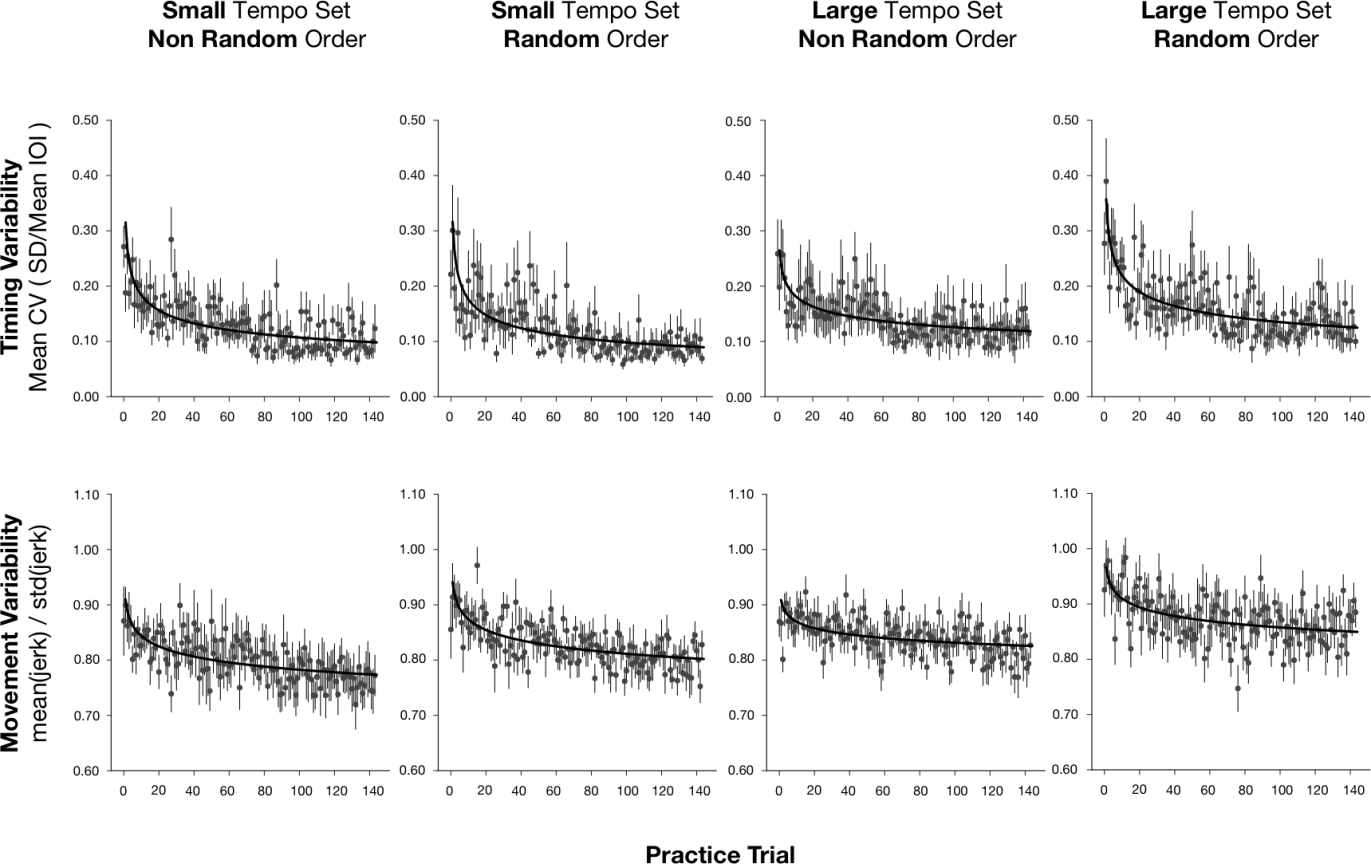
Mean timing and movement variability for the practice trials. Top row: Mean timing coefficients of variation for the 144 practice trials, with standard errors for each trial, reported for each practice group by Tempo Set and Order. Bottom row: Mean movement smoothness values for the 144 practice trials with standard errors for each trial, reported for each practice group.

We assessed non-musicians' timing variability during learning trials with the coefficient of variation (CV) of the IOIs. Timing variability was expected to decrease in all conditions during practice. Fig 4 (top row) shows the mean CV by learning trials for each group.

To assess the evolution of skill development during practice, we measured the learning rate by the slope of performance plotted against learning trial for each tempo and ordering conditions. Improvement over practice is usually assessed through a power law; the logarithm of task performance plotted against the logarithm of the number trials usually yields a straight line [27,28]. The evolution of task performance with practice described by the general power law is given by the equation: *Y = A+B(T+E)^-α^*, where *Y* denotes the CV values; *A* is the asymptotic value of performance; *B* is the task performance on the first trial; *T* is the number of trials; *E* is the initial expertise (prior to the first trial); and *α* is the learning rate.

To assess if the decrease in timing variability during practice depended on the tempo and order conditions, we fit the general power law to the individual CV measures for each tempo and order condition. The power law model provided a significant fit for 37 participants out of 48 (M(r) = -0.28, SE = 0.04). The powerlaw decay is negative for 36 participants meaning an improvement in practice. The fit of the power law, represented by *r*, was entered in a two-way ANOVA by Tempo Set and Order; there were no significant main effects or interaction, indicating the powerlaw fit did not differ across the practice conditions. The learning rate, represented by the decay exponent *α*, was also evaluated with a two-way ANOVA with Tempo Set (2 or 6), and Order (Random or non-Random) as factors. There was no significant effect of Tempo Set (F(1,44) = 2.20, p = 0.15, *η*^2^ = 0.05) or Order (F(1,44) = 0.39, p = 0.54, *η*^2^ = 0.01), or interaction between factors, indicating that the learning rates were similar across conditions.

Similarly, we assessed motor skill acquisition with change in movement smoothness during practice. Fig 4 (bottom row) shows the mean movement smoothness across practice trials for each of the 4 practice groups. We fit the general power law to the individual movement smoothness measures for each tempo and order condition. The power law model provided a significant fit for 30 participants out of 48 (M(r) = -0.20, SE = 0.02). The powerlaw decay is negative for 29 participants meaning a decrease of movement variability. Individual decay coefficients (learning rates) were evaluated with a two-way ANOVA with Tempo Set and Order as between-subject factors; there were no significant effects of Tempo Set or Order and no interaction, indicating that the learning rates of motor skill acquisition did not differ across conditions.

**Fig 5:**
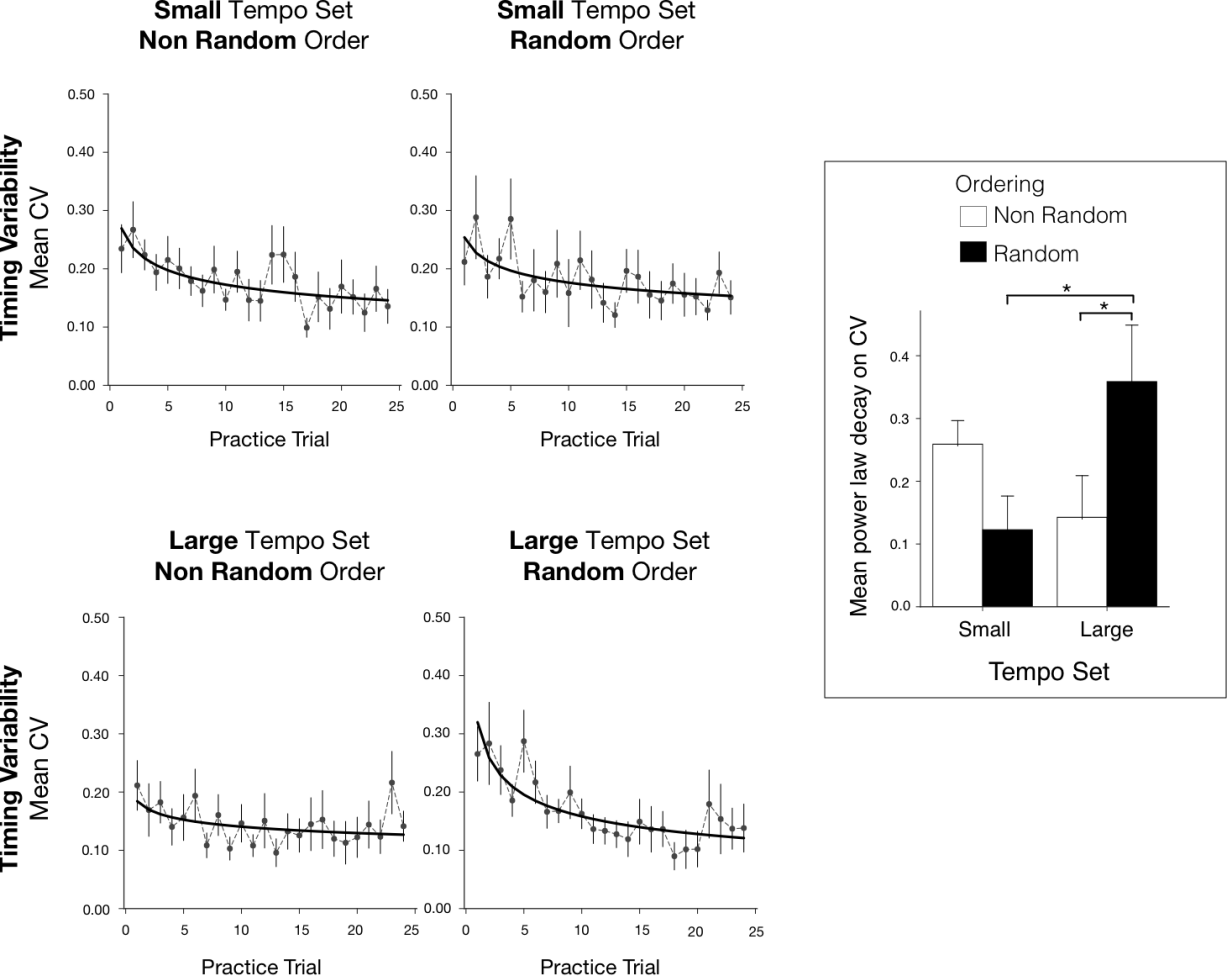
Mean timing coefficients of variation and learning rates. Mean timing coefficients of variation and fit of power law for the first 24 learning trials at the specific tempo 350ms, with standard error bars, by Tempo Set and Order. Right panel: Mean learning rates from power law fit to individual data with standard error bars, applied to coefficients of variation from the first 24 learning trials at 350ms by Tempo Set and Order.

All groups practiced the tempo 350ms during practice and this tempo was also tested for all groups after practice. Therefore, we analyzed whether learning rates on this specific tempo varied across practice conditions. Since two groups practiced 24 learning trials at 350ms (Large Tempo Set condition) and two other groups practiced 72 learning trials at 350ms (Small Tempo Set condition), we compared the 24 first learning trials at 350ms across the four groups. Fig 5 shows the mean CV by learning trials and group.

We examined whether the learning rates differed across groups by performing a two-way ANOVA by Tempo Set and Order, on the mean decay parameters computed on the timing CV for the first 24 learning trials at tempo 350ms. There were no significant effects of Tempo Set (F(1,44) = 2.20, p = 0.15, *η*^2^ = 0.05) or Order (F(1,44) = 0.39, p = 0.54, *η*^2^ = 0.01), but there was a significant interaction between Tempo Set and Order (F(1,44) = 6.8, p < 0.05, *η*^2^ = 0.13). Post-hoc analyses indicated greater learning rates in the Large, Random order tempo set than in either the Small, Random order or the Large, Non Random order tempo sets (Tukey HSD = 0.13, p < 0.05). To analyze if this effect is due to differences in performance at the first learning trial at 350ms, we assessed whether the mean CV differed across practice groups at that trial with a two-way ANOVA by Tempo Set and Order. The analysis indicated no main effect of either factor (F(1,44) = 0.004, p = 0.95 for Tempo Set and F(1,44) = 0.13, p = 0.73 for Order) and no interaction (F(1,44) = 0.49, p = .49) on data from the first learning trial. We repeated the analysis on data at the 24th learning trial at 350ms. A similar two-way ANOVA also indicated no main effect of either factor (F(1,44) = 2.35, p = 0.13 for Tempo Set and F(1,44) = 0.14, p = 0.72 for Order) and no interaction (F(1,44) = 0.81, p = 0.37). Therefore, the timing variability did not differ across groups at the beginning of practice at tempo 350ms or at the end of the 24 trials.

### Transfer of tempo

#### Performance at test tempo 350ms, practiced during training

**Fig 6:**
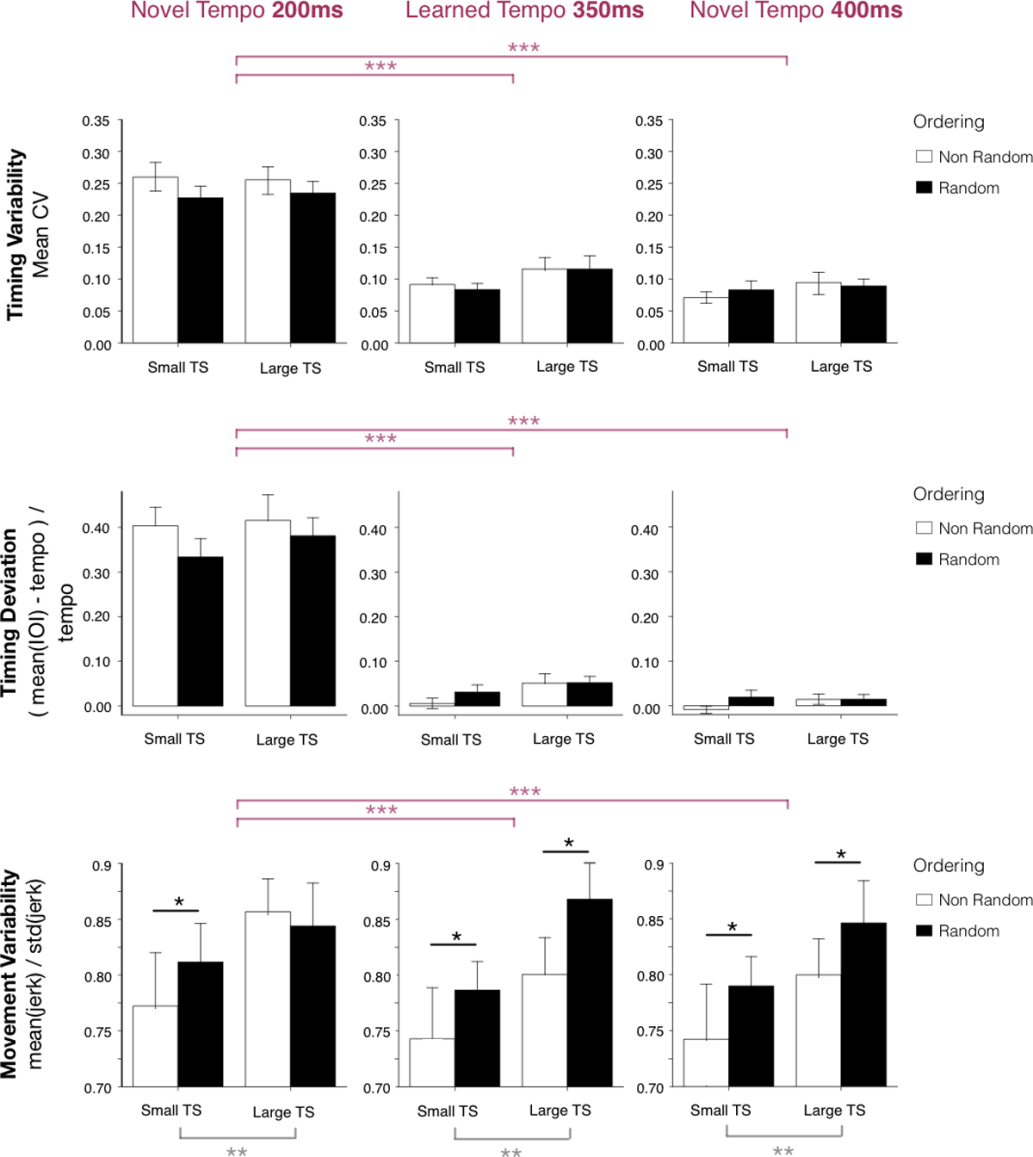
Tempo transfer tests. Top row: Mean timing coefficients of variation with standard error bars by Tempo Set and Order, for each test tempo: novel tempo 200ms (left), learned tempo 350ms (middle), and novel tempo 400ms (right). Middle row: Mean signed timing deviation with standard error bars by Tempo Set and Order, for each test tempo. Bottom row: Mean movement smoothness with standard error bars by Tempo Set and Order, for each test tempo.

Performance at test was first compared on the 350ms rate test melody that all learning groups performed. A two-way ANOVA on the CVs for the 350ms melody performed at test with between-subjects factors of Tempo Set and Order revealed no significant effect of factor Order (F(1,44) = 0.67, p = 0.80) and a marginal effect of Tempo Set (F(1,44) = 3.28, p = 0.08, *η*^2^ = 0.07). Mean timing CVs tend to be higher for the Large Tempo Set condition. There was no interaction between factors. Similarly, we repeated the analysis on the signed timing deviation measures (observed IOIs minus the expected IOIs, divided by the expected IOIs) computed for each finger keystroke. Positive deviation measures mean lengthened intervals and negative values mean shortened intervals. The analysis revealed no significant effect of Order (F(1,44) = 0.59, p = 0.45), a marginal effect of Tempo Set (F(1,44) = 3.8, p = 0.06, *η*^2^ = 0.08), and no interaction between factors. Mean timing accuracies tended to be closer to 0 for the Small Tempo Set condition and more negative for the Large Tempo Set condition. This is depicted in Fig 6, middle row, middle panel.

We then assessed whether movement variability at test tempo 350ms differed across practice conditions. A two-way ANOVA showed a significant effect of Tempo Set (F(1,44) = 7.8, p < 0.01, *η*^2^ = 0.15) and Order (F(1,44) = 5.4, p < 0.05, *η*^2^ = 0.11) with no interaction. As shown in Fig 6 (bottom row, middle panel), movement variability was higher in the Large Tempo Set than Small Tempo Set conditions (p < 0.01) and higher for the Random than the non-Random Tempo Order (p < 0.05).

#### Transfer to averaged tempo 400ms and fast tempo 200ms

We next assessed whether the practice conditions affected timing variability at transfer to different tempi (400ms and 200ms) compared to the test at the familiar 350ms tempo. Mean CVs at test were evaluated with a three-way analysis of variance with Tempo Set and Order as between-subjects factors and Test Tempo as within-subjects factor. Test Tempo had a significant effect (F(2,44) = 192.4, p < 0.001, *η*^2^ = 0.81); there was no main effect of Tempo Set or Order, or interaction between factors. As shown in Fig 6 (top row), mean CVs were higher for test tempo 200ms (fast novel transfer tempo), than for the 400ms (slow novel transfer tempo) and 350ms (previously learned tempo). Similarly, we assessed whether the mean signed timing deviation differs across the test tempi (practiced tempo 350ms and two transfer tempi 400ms, 200ms) by practice conditions. We found a significant main effect of Test Tempo (F (2,44) = 556.8, p < 0.001, *η*^2^ = 0.93), no effect of tempo Order or Tempo Set, and no interactions. Pairwise t-tests with Bonferroni corrections revealed that mean timing deviation is lower at transfer test tempo 400ms than control tempo 350ms (t = -17.2, p<0.001) and transfer tempo 200ms (t = -26.5, p<0.001), and the mean timing deviation is lower at 350ms than 200ms (t = -20.5, p<0.001). In other words, timing accuracy is better (signed deviation closer to 0) for slower tempo 400ms, followed by 350ms and 200ms. This is shown in Fig 6, middle row.

We then repeated the analysis on movement variability. Mean variability measures were analysed with a three-way ANOVA with between-subjects factors Tempo Set and Order and within-subjects factors Test Tempo (the trained tempo 350ms and two transfer tempi 400ms and 200ms). There was a main effect of Tempo Set (F(1,44) = 13.2, p<0.01, *η*^2^ = 0.23), with higher variability for the Large than Small tempo set condition.

There was also a main effect of Order (F(1,44) = 5.2, p<0.05, *η*^2^ = 0.11) with higher variability for the Randomized condition than Non-randomized (see Fig 6 bottom row). The main effect of Test Tempo was also significant (F(2,44) = 4.6, p<0.05, *η*^2^ = 0.10) and there was no interaction with other factors. Similar to the findings for timing variability, movement variability was higher at Test Tempo 200ms than at 400ms (t = -2.4, p<0.05) and 350ms (t = -2.1, p<0.05). There was no significant difference between 350ms and 400ms tempi.

### Transfer of sequence

**Fig 7:**
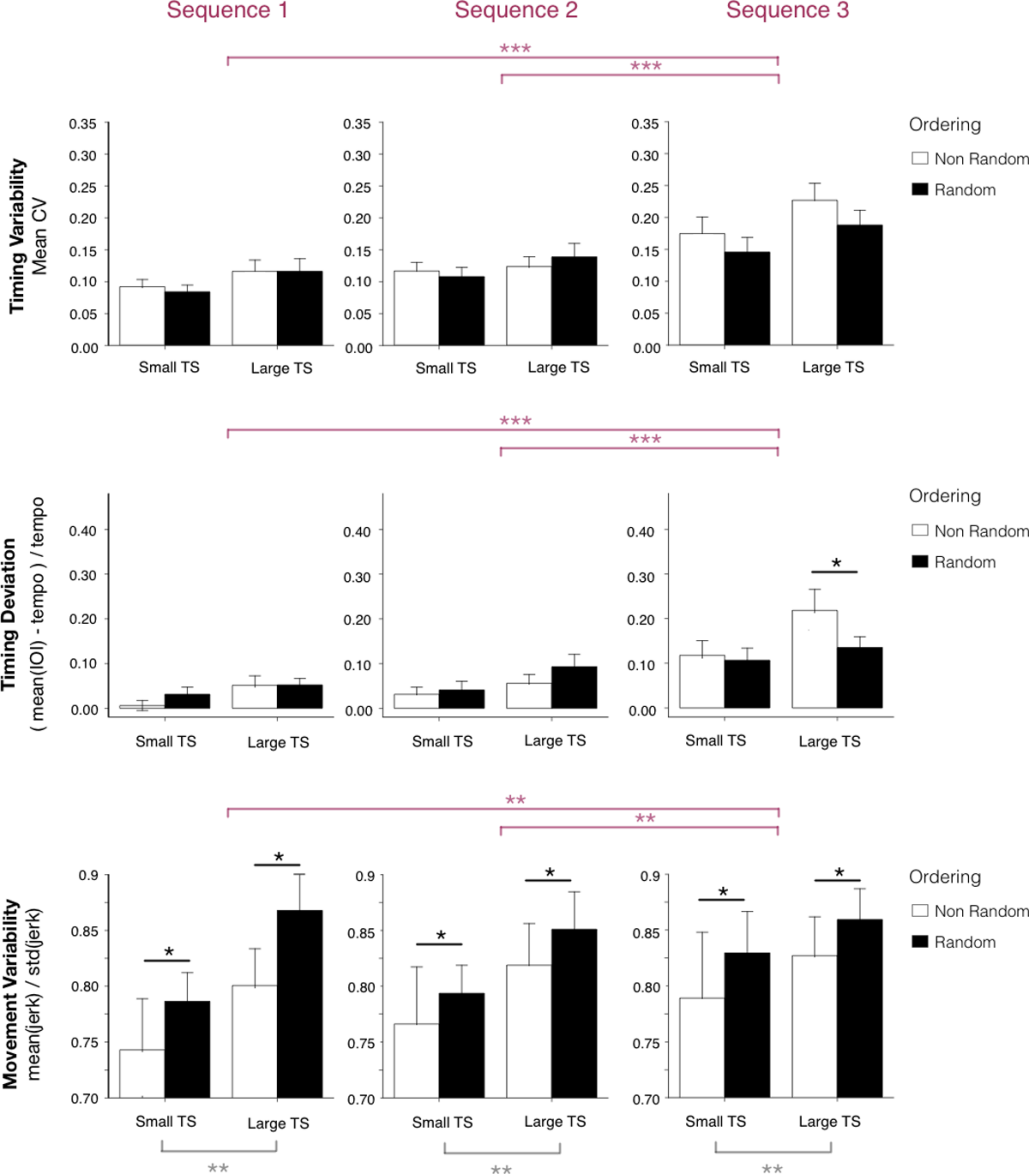
Transfer test sequences. Top row: Mean timing coefficients of variation with standard error bars by Tempo Set and Order, for practiced Sequence 1 (at 350ms), transfer Sequences 2 and 3 (performed at 350ms). Middle row: Mean signed timing deviation with standard error bars by Tempo Set and Order, for each test sequence. Bottom row: Mean movement smoothness with standard errors by Tempo Set and Order, for each test sequence.

We examined the performance when transferring to novel sequences. We assessed whether the temporal variability differs when transferring to two new sequences, transfer sequences 2 and 3, compared to the practiced sequence. We compared the timing variability in transfer sequences performed at tempo 350ms following practice of Sequence 1 at the same tempo 350ms. Mean CVs were evaluated with an ANOVA on Tempo Set and Order as between-subjects factors and Sequence (sequences 1, 2 and 3) as a within-subjects factor. Only the main effect of Sequence was significant (F(2,44) = 45.4, p < 0.001, *η*^2^ = 0.51); as shown in Fig 7 (top row), the mean CVs were higher for Sequence 3 than for Sequences 1 and 2 (t = +0.085, p<0.001 and t = +0.059, p<0.001 respectively).

We repeated the analysis to test the signed timing deviation values when transferring to the new sequences Sequence 2 and 3, compared to Sequence 1. The analysis revealed a main effect of Sequence (F(2,44) = 46.8, p < 0.001, *η*^2^ = 0.52) and an interaction between Sequence and Order (F(2,44) = 5.0, p < 0.05, *η*^2^ = 0.1). Post-hoc analyses indicated better timing accuracy for Sequence 1 and 2 than Sequence 3 (the sequence with most movement changes from Sequence 1), and better timing accuracy in the Random order than Non-Random order for Sequence 3 (Tukey HSD = 0.031, p < 0.05). The main effect of the practice condition Tempo Set was significant (F(1,44) = 4.5, p < 0.05, *η*^2^ = 0.09). See Fig 7, middle row. A post-hoc analysis with Bonferroni correction revealed that the mean timing deviations are lower for the Small Tempo Set condition than Large Tempo Set (t = 2.98, p<0.01). There was no interaction between factors.

Finally, we performed the analysis of sequence transfer effects on the movement variability measures. A three-way ANOVA was applied to movement variability measures with between-subjects factors Tempo Set and Order and within-subjects factors Sequence. We found a main effect of Sequence (F(2,44) = 5.2, p<0.01, *η*^2^ = 0.11). As shown in Fig 7 (bottom row), movement variability was higher for transfer sequence 3 than transfer sequence 2 and the original sequence 1. There was also a main effect of the Tempo Set (F(1,44) = 8.4, p<0.01, *η*^2^ = 0.16), with higher variability for the Large than the Small tempo set condition. Finally, there was a main effect of Order (F(1,44) = 4.9, p<0.05, *η*^2^ = 0.10) with higher variability for the Randomized condition than the Non-randomized condition. There were no interactions between variables.

### Correlations between timing and movement analyses

In the analysis below, we computed simple correlations between two datasets from which we discarded individual outliers (values greater than 3 SD from the mean). We first tested if the decay functions for the power law fit to the movement smoothness values from the practice trials were related to the decay functions from the timing (CV) values from the practice trials within the same individuals. We found that there is no correlation (r = -0.14, p = 0.52) between the motor and timing learning rates.

**Fig 8:**
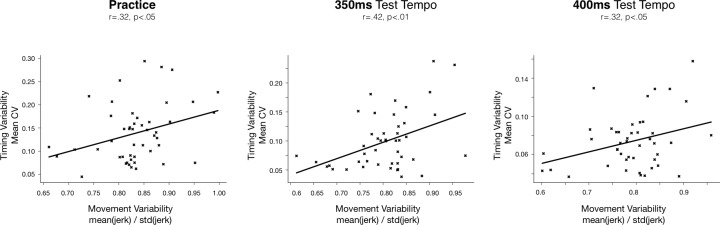
Correlations between timing and movement variability. Inter-individuals' correlation between the mean movement variability (movement smoothness) and the mean timing variability (timing IOI CVs) computed across the practice phase (left panel), test at practiced tempo 350ms (middle panel), and transfer tempo 400ms (panel right).

Then we tested the correlations between movement and timing variability measures (smoothness and CVs respectively) during each phase of the study: practice phase, test phases at 350ms, 400ms, 200ms and test phases with sequence 2 and 3. Pearson correlation coefficients were computed at each phase, after outliers were discarded (n = 0 at practice, n = 2 at 350ms, n = 3 at 400ms), and we found a significant positive correlation only in practice phase (r = 0.32, p < 0.05), at test tempo 350ms (r = 0.42, p<0.01) and transfer tempo 400ms (r = 0.32, p<0.05). This is depicted in Fig 8.

## Discussion

This study examined the role of temporal variations in a musical motor task on nonmusicians' learning and transfer of timed movement sequences. Overall, the findings indicated dissociable effects of temporal variation during practice on motor and timing skill acquisition. Timing skill acquisition, as measured at test, was not affected by the practice conditions (number of different tempi practiced or scheduling of these tempi), although there was a significant beneficial effect of temporal variation on learning rates during practice at specific tempo (350ms IOI). In addition, the larger number of different tempi practiced and higher contextual interference from interleaved tempi impaired motor skill learning, measured by movement variability. Thus, the systematic benefit of practicing variations of a motor task on transfer of learning to related motor tasks [2,4] does not extend to timed movement sequences typical of music. The following sections review these findings and propose interpretation with regard to previous work.

First, we showed that learning rates during practice, based on coefficients of variation (measures of inter-keystroke interval variability) and measures of jerk (movement smoothness), were well fit by a power law of learning for the majority of participants. This is consistent with previous research on law of practice in skill acquisition [27,28]. These fits did not show different learning rates overall when compared across different practice tempi and their ordering. However, comparison of learning rates for the tempo condition common to both the large and small tempo sets (350ms IOI) yielded significant differences in learning rate, with an interaction between the size of tempo set and tempo ordering in temporal variability measures (CV). This interaction indicated an advantage (steeper learning rate) for the 350ms tempo condition when it was presented in a large tempo set in random order, both findings consistent with previous literature on motor learning [4]. Thus, pianists' performance timing showed differential improvement during learning consistent with contextual interference and large motor variability at practice, but only within a common tempo and not across changing tempi.

Second, each individual's timing variability and motor variability showed significant correspondence, both at practice and at test. These findings confirm the use of coefficients of variation and smoothness (jerk) as appropriate indices of learning in the music performance task. The two measures of variability were correlated for both the practiced tempo and the mid-range (350 ms) transfer tempo, with the highest correlation at test for the practiced tempo (deviations from the correct tempo were related to the variability in movement execution). The fact that the timing-motor variability correspondence reached its maximum for the practiced tempo, and decreased or became null otherwise, suggests that both types of variability become more correlated with practice at a specific tempo.

Third, different practice conditions (number of tempi and tempo order) had consistent and significant effects on motor variability at test, for both previously practiced and novel tempi. The Small Tempo Set and Non-random ordering led to smaller motor variability at test, suggesting that the musical motor skill showed greater generalization following learning conditions with smaller temporal variation and non-random ordering. This finding, that may appear in contrast with previous research showing that variable practice improves transfer of learning of a motor task [2,4,12], can be interpreted by the fact that variability of practice and contextual interference have led to poorer performance at immediate test (on familiar and transfer tasks) whereas better performance has been observed at retention, usually assessed few hours (even few days) after training [4,5,8]. Interestingly, sequence timing variability at test was unaffected by the order of tempi or the number of different tempi practiced. This suggests that the effects of contextual interference and variability of practice may not apply similarly to timing and motor skill learning. This dissociable effect may be due to different underlying computational mechanisms: motor learning is usually associated with a combination of error-based processes adapting an internal model inferring motor commands [29], and reward-based processes allowing for exploration and exploitation of motor commands producing the maximum long-term reward [30]. In contrast, timing acquisition, considered as the synchronization of a movement to an external stimulus, is usually seen as to the coupling of non-linear dynamical systems in order to maintain a stable phase relationship [31].

Finally, task variability during practice facilitated transfer to novel sequences that differed in movement difficulty (spatial manipulation). For both novel sequences 2 and 3 (designed to have greater movement difficulty than the learned sequence), there were significant effects of tempo order during practice on the timing accuracy at transfer. Randomized tempo orders improved timing accuracy for the novel test sequences, consistent with the predictions of contextual interference. In contrast, motor variability at test was negatively affected by the number of different tempi (the amount of variability) present during the practice conditions: practice with large tempo sets led to greater motor variability at test. Previous research on spatial variations during learning found that at immediate test after training, there is no benefit of practice at test for higher amounts of spatial variability [5,32,33], consistent with our findings. Our results explicitly showed that lower temporal variations of a motor task may induce greater generalization to other motor tasks that involve different levels of motor complexity. In other words, contextual interference seems to have a slight benefit on timing accuracy in transfer of sequences but its effect does not generalize to timing variability and motor variability.

The limited benefits of the amount and the schedule of task variability at practice on motor learning transfer to novel tempi and novel sequences may be linked to the expertise of the participants. Previous studies have shown the limitations of variability of practice and contextual interference in ecological situations, such as volleyball serve [34], suggesting that variability in learning schedule may be linked to the level of expertise in a given skill [13]: higher skilled individuals may benefit more from this type of practice than novices. Our findings support this interpretation in the context of a musical task. In fact, in piano task performed by expert pianists, practicing at a submaximal speed a given phrase increases the maximal speed of the execution of the same phrase [35]. However, our findings showed that, in the case of novices, practicing a sequence at 250ms or 300ms does not improve the performance at fast tempo 200ms.

Future studies may address the perceptibility of timing and motor variations as a possible explanation for differential learning effects on transfer. Previous work has shown that the range of motor variation may have different effects on learning, depending on how the variations are perceived by the learner [36]. The influence of motor variations on transfer was investigated as people learned a new walking pattern, and variations were experienced as errors that the learner tried to correct or minimize. A small spatial error range (within the natural human range) was experienced as endogenous error (arising from within the body) that aided transfer, while a large error range was experienced as exogenous (coming from the environment) and limited transfer to a new walking pattern [36]. We should note that their task was familiar to participants (walking) and was manipulated by creating unusual patterns. In our study, the task was novel for all participants. In our study, the large range of (novel) variability could act as a limiting factor for the transfer to new sequences and tempi; an interesting extension would be to examine the role of perceptibility thresholds for temporal variability that change as a function of music skill learning.

In sum, we tested the effects of task variability (by manipulating tempo set size and tempo ordering) on temporal learning (timing variability and accuracy) and motor learning (movement variability) in nonmusicians' transfer to novel and familiar tempi and sequences. We hypothesized that higher variability during practice and higher contextual interference (randomized tempo orders) would affect motor variability at test but not timing variability. The findings revealed that higher tempo variability during practice induced poorer performance in motor learning at immediate test, whereas timing variability at test was not affected. This confirms a dissociable effect of motor task variations on timing and motor skill acquisition at the earliest stages of practice, in line with previous theoretical research on the link between timing and movement in music performance [19,20,37,38]. These findings extend our knowledge of skill acquisition to a novel population (beginners) with confirming measures from motion and timing.
